# The Story of "Sister Olive"

**Published:** 1887-01-08

**Authors:** 


### THE STORY OF "SISTER OLIVE."

May I, as a nurse of many years' experience, be permitted to say a few words in the "Sister Olive" controversy, caused by the letters of Miss Wood and Miss Twining? I cannot but think that those ladies have formed ideas of propriety more suited to a French convent than to any section of English society, where in every class men and women are accustomed to mingle freely from their earliest to their latest years. Are we to infer that ladies and gentlemen who are engaged, often from the highest motives, in hospital work, are the only people unworthy of the freedom enjoyed by all others? I can thoroughly sympathise with the difficulties that must always occur in maintaining discipline amongst a large body of workers, embracing infinite varieties of character; but I do not think that martinets are the best disciplinarians. On the contrary, I believe that nothing is more destructive of the true spirit of discipline than a system of arbitrary and needless restriction. Wherever quick feelings and wide sympathies exist, they are sure to seek an outlet, and the perhaps fortunate persons who do not possess those qualities are better fitted for the manipulation of dead matter of any sort, than for tending the suffering compounds of body, soul, and spirit called patients. I will only say, in conclusion, that I consider the spirit that runs through "Sister Olive" as much above the morality of its critics as the free spirit of Christian ethics exceeds the righteousness of the Scribes and Pharisees.

Salop. An Englishwoman.

